# Cannabis legalization in the US. Where do we go from here?

**DOI:** 10.47626/2237-6089-2022-0001

**Published:** 2022-06-27

**Authors:** Silvia S. Martins, Natalie S. Levy, Emilie Bruzelius, Luis E. Segura

**Affiliations:** 1 Department of Epidemiology Mailman School of Public Health New York NY USA Department of Epidemiology, Mailman School of Public Health, New York, NY, USA.

Cannabis is the most commonly used illegal drug globally.^1-4^ Use often begins in adolescence^5-7^ and heavy adolescent and young adult cannabis use is associated with serious negative consequences.^5,6,8-11^ For example, longitudinal studies show that weekly cannabis use and development of abuse or dependence in adolescence are associated with increased risk of school dropout, truancy, and unemployment.^10^ Cannabis use has also been associated with depression, anxiety disorder, and suicidal ideation across the life course, and may be associated with other adverse outcomes in certain populations or settings.^12-15^

Cannabis use was legal in the United States (US) in the 1800s and was commonly used therapeutically.^16-18^ However, following the development of synthetic painkillers^19^ and a period of considerable media attention to cannabis-related violence, Congress outlawed recreational use in 1937 and made access for medical use burdensome.^16,17,19^ In 1970, cannabis was defined federally as a Schedule I substance with “no…accepted medical use” effectively making all cannabis use illegal.^20^ However, in 1996, California passed Proposition 215, allowing for medical use of cannabis in the state.^21^ Since then, cannabis laws have changed rapidly across the US. As of January 2022, 18 states and Washington (DC) had fully legalized cannabis use for adults aged ≥ 21 years and an additional 18 states had legalized medical cannabis.^22^

Opponents of cannabis legalization suggest that it would increase the availability of cannabis, making its use and attendant negative consequences more widespread.^23^ The possibility of increased cannabis use among youth is of particular concern. In this article we will present evidence from several of our research group’s studies evaluating the relationship between enactment of medical and/or recreational cannabis laws and cannabis use outcomes in the US. We will also briefly present data from other research teams on the role of cannabis dispensaries in changing patterns of cannabis use.

## Recent trends in cannabis use in the US overall and by cannabis legalization status

Our work has identified differing trends in cannabis use by demographic characteristics. While cannabis use is generally more common among men than women,^24^ the prevalence of use increased for both groups from 2002 to 2014. However, we further found that increases were greater for men (+4.0%) than for women (+2.7%), leading to a widening of the gender gap over time (p < 0.001). This divergence was primarily driven by increased prevalence among men in the lowest income level (+6.2%) from 2007 to 2014.^25^ Among women, trends also suggest increased cannabis use during pregnancy. We found that between 2002-2014, past month use among pregnant women increased 62% nationally.^26^

Our work has also uncovered age differences in cannabis use, including significant increases in the prevalence of past-year cannabis use among older adults from 2006/07 to 2012/13, with a 57.8% relative increase for adults aged 50-64 (linear trend P < 0.001) and a 250% relative increase for those aged ≥ 65 (linear trend P = 0.002).^27^ We have also found that daily cannabis use prevalence increased significantly in 2014 versus 2007 for all age groups 12 years and older and no differences in the absolute increase by age group were observed. In contrast, for non-daily cannabis use, absolute increases in the prevalence of use among adults 26-34 (+4.49 percentage points) were significantly larger than increases in all other ages except 50-64 (+3.02 percentage points). From 2002-2014, middle-aged adults 50-64 reported the largest increases in non-daily cannabis use (+4.37 percentage points).^28^

## Medical cannabis laws and cannabis use outcomes

Using National Surveys of Drug Use and Health (NSDUH) restricted-use individual-level data, we found that from 2004 to 2013, states that passed medical cannabis laws had higher prevalence of past-month cannabis use before passing medical cannabis laws compared with states that never passed these laws.^29^ We also found evidence of increases in both the prevalence of past-month cannabis use and the prevalence of perceiving cannabis as easily available after the passage of medical cannabis laws among those 26 and older. Comparing the period after passage of medical cannabis laws with the period before passage, the adjusted odds ratio (aOR) for past-month cannabis use was 1.24 (95% confidence interval [95%CI] 1.16-1.31) and for perceived availability was 1.11 (95%CI 1.07-1.15). However, no differences in prevalence were observed for younger age groups.

We have also used NSDUH data to examine whether the age-specific impact of medical cannabis laws on the prevalence of cannabis use, daily cannabis use, and past-year cannabis use disorder varies by gender. We found that among those 26+, past-month cannabis use increased significantly for men from 7.0% before to 8.7% after enactment of medical cannabis laws (+1.7%, p < 0.001) and for women from 3.1% before to 4.3% after enactment (+1.1%, p = 0.013). In this age group, daily cannabis use among those reporting past-month cannabis use also increased significantly after enactment for both genders (men: 16.3% to 19.1%, +2.8 %, p = 0.014; women: 9.2% to 12.7%, +3.4%, p = 0.003). We did not observe any significant increases in past-year cannabis use disorder prevalence for any age or gender group after medical cannabis law enactment.^30^

The empirical findings from our studies were also supported by a systematic review of articles testing quantitative differences in cannabis use among 10 to 25-year-olds following liberalization of cannabis policy (decriminalization and legalization).^31^ Two independent readers identified 41 original research reports for review. The highest quality studies found no evidence of a change in medical cannabis use following cannabis decriminalization or legalization for medical purposes. A slight increase in recreational use was observed for adolescents and youth after recreational legalization.

An implicit assumption of all studies investigating the effects of medical cannabis laws on cannabis use is that all individuals residing in a state are aware of state policies and can adjust their behavior accordingly. We interrogated this assumption and found that from 2004-2013, an average of 68.6% of individuals in states that never passed medical cannabis laws correctly identified that their state had not legalized cannabis for medical use. In states that ever passed a medical cannabis law, 67.7% correctly identified that their state did not have medical cannabis laws before medical cannabis law enactment, while only 44.8% correctly identified that their state had a medical cannabis law after enactment of the law.^32^ We also found that adolescents had lower medical cannabis law knowledge than adults, consistent with earlier findings that the effects of medical cannabis law on cannabis use were restricted to adults only.

Our research has also explored how enactment of different types of medical cannabis law programs impact subsequent patterns of cannabis use. We compared changes in the prevalence of cannabis use, frequent cannabis use, and cannabis use disorder in states with highly regulated (“medicalized”) medical cannabis law programs to those in states with “non-medical” medical cannabis law programs. We found that more loosely-regulated medical cannabis law programs were associated with increases in past-month cannabis use and frequent cannabis use among adults 26+ after medical cannabis laws were enacted. No increases in cannabis use prevalence were observed in states with more highly regulated medical cannabis law programs. Additionally, there were no increases in adolescent or young adult cannabis use outcomes following medical cannabis law passage, irrespective of program type.^33^

Relatedly, we also explored how the stringency of medical cannabis law regulations affects enrollment rates on medical cannabis programs. We found that fourteen of the twenty-four programs assessed were nonmedical (more loosely regulated) and collectively enrolled 99.4 percent of all participants nationwide, with enrollment rates twenty times greater than programs deemed to be “medicalized”. These results suggested that policy makers implementing or amending medical cannabis programs should consider the powerful relationship between less regulation and greater enrollment.^34^ More research is needed to determine the relationship between program enrollment, variation in medical cannabis indications, and cannabis-related outcomes.

The broader state policy context may also modify the effects of medical cannabis laws on cannabis use and cannabis use disorder. We examined the association between state-level policy liberalism and past-year cannabis use and cannabis use disorder for individuals aged 12-17, 18-25, and 26+. In adjusted models, liberal states had higher average past-year cannabis use than conservative states for ages 12-17 (+1.58 percentage points; p = 0.03) and 18-25 (+2.96 percentage points; p = 0.01). However, cannabis use disorder among those using cannabis was lower in liberal states compared to conservative states for ages 12-17 (-2.87 percentage points; p = 0.045) and ages 26+ (-2.45 percentage points; p = 0.05).^35^

## Medical cannabis laws, other substance use outcomes, and traffic fatalities

Our team has also performed several analyses examining how passage of state medical cannabis laws might result in changes in the use of other substances. For example, it has been suggested that increased availability of cannabis might play a role in tackling the ongoing opioid epidemic, perhaps through substitution. To that end, we have conducted several studies exploring the relationship between medical cannabis laws and prescription opioid use. Overall, our results do not support any changes in nonmedical prescription opioid use or prescription opioid use disorder^36^ nor in fatal opioid overdose^37^ associated with medical cannabis laws. We did find some evidence that medical cannabis laws authorizing home cultivation or dispensaries are associated with reductions in opioid positivity among 21 to 40-year-old fatally injured drivers, but no overall significant association was found.^38^ These findings call into question the idea that medical cannabis laws may have a protective effect on prescription opioid use and overdoses, suggesting that other solutions to the opioid crisis are needed.

We have also studied the effects of medical cannabis laws on the direct consequences of cannabis use, particularly traffic fatalities. Using data from the 1985-2014 Fatality Analysis Reporting System (FARS), we examined the association between medical cannabis laws and traffic fatalities, while controlling for contemporaneous secular trends. We found that on average, states with medical cannabis laws in place had lower traffic fatality rates than states without such laws. Medical cannabis laws were associated with reductions in traffic fatalities in those aged 15-24 and 25-44 years immediately after passage and with ongoing albeit weaker reductions in those aged 25-44 years in the years after enactment of medical cannabis laws. The presence of dispensaries in a state was also associated with traffic fatality reductions in those aged 25-44 years.^39^

## Recreational cannabis laws and cannabis use outcomes

More recently, our team has turned its attention to expanding the limited available research on the effects of recreational cannabis laws on cannabis use outcomes. Using 2008-2016 NSDUH data, we found that similarly to medical cannabis laws, changes in cannabis outcomes were primarily only observed for those 26 years of age and older. Following recreational cannabis law enactment, the population prevalence of past-month cannabis use among those 26+ increased from 5.65% to 7.10% (aOR compared with states without recreational cannabis laws: 1.28; 95%CI 1.16-1.40), past-month frequent use increased from 2.13% to 2.62% (aOR: 1.24; 95%CI 1.08-1.41), and past-year cannabis use disorder increased from 0.90% to 1.23% (aOR: 1.36; 95%CI 1.08-1.71).^40^ However, when restricting our analysis to individuals 26+ who reported using cannabis, no changes in frequent use or cannabis use disorder were observed. This suggests that there are other explanations for the small observed increases in the adult population prevalence of frequent use and cannabis use disorder, for example positive secular trends in the prevalence of cannabis use preceding legalization,^41^ rather than legalization leading to increases in problematic use among those using cannabis. Indeed, as reported in other work, the population prevalence of cannabis use disorder declined from 2002-2017, from 14.8% to 9.3% among adults reporting any past-year cannabis use and from 33.4% to 19.5% among adults who used cannabis daily/near daily.^42^

In this same study, no meaningful changes in cannabis use outcomes were found among respondents aged 18 to 25 years. A small increase in the population prevalence of past-year cannabis use disorder was observed for those 12-17 years of age, from 2.18% before to 2.72% after recreational cannabis law enactment and this represented a 25% higher increase compared with 12 to 17-year-olds in states that did not enact recreational cannabis laws (aOR: 1.25; 95%CI 1.01-1.55). Among 12 to 17-year-olds reporting past-year cannabis use in this age group, cannabis use disorder increased from 22.80% before enactment to 27.20% after (aOR: 1.27; 95%CI 1.01-1.59). While the significant association between recreational cannabis laws and cannabis use disorder in this age group warrants additional interrogation, the increasing prevalence of cannabis use disorder among those 12-17 years of age is concerning regardless of its cause and should continue to be monitored.

We have also pursued studies exploring how patterns of cannabis use following recreational cannabis law enactment may differ by race and ethnicity. We found that compared with the period before recreational cannabis law enactment and after medical cannabis enactment, the odds of past-year cannabis use after enactment increased among Hispanic (aOR: 1.33; 95%CI 1.15-1.52), non-Hispanic other race (aOR: 1.31; 95%CI 1.12-1.52), and non-Hispanic White (aOR: 1.21; 95%CI 1.12-1.31) populations, particularly among those aged 21+. Similar increases were observed in the odds of past-month cannabis use among these same populations. However, we found no increases in the odds of past-year or past-month cannabis use among non-Hispanic Black individuals, nor among individuals aged 12-20 years regardless of race/ethnicity.^43^ Our study was the first study to explore racial/ethnic-specific and age-stratified associations with recreational cannabis laws, particularly focusing on the period after enactment of preexisting medical cannabis laws. Separating out the effects of these laws is of utmost importance since studies conducted prior to recreational cannabis law enactment identified differential trends in cannabis outcomes over time by race/ethnicity. In an effort to minimize racial inequalities in cannabis legislation enforcement, future studies will need to examine patterns of cannabis use in the context of persistent racial/ethnic disparities in cannabis arrests and incarceration.^44,45^

## Cannabis dispensaries and cannabis use

A growing body of research focuses on intended and unintended effects of cannabis use, consequences of cannabis availability, and the effects of dispensary programs on local communities. Interestingly, increased density of dispensaries has been associated with increased cannabis hospitalizations,^46^ poison center calls,^47^ and a higher likelihood of adolescent cannabis use.^48^ Budney and Borodovsky^49^ argue for more stringent regulations related to levels of access created by medical and recreational dispensaries, particularly cannabis product potency and how cannabis products are consumed. As more states adopt recreational cannabis laws and more time has passed since their enactment, additional studies will be needed to explore the persistent effects of legalization.

## Limitations

The studies described above are not without limitations. First, almost all of the analyses described here relied on self-reported cannabis use and the social desirability of reporting cannabis use and consequent measurement error may have changed differentially by state medical and recreational cannabis law status. In the NSDUH, the dataset used for most of the studies described here, the use of computer-assisted interviews reduces these concerns.^50^ Second, the NSDUH also excludes homeless individuals who do not live in shelters, individuals residing in institutions, and incarcerated individuals, which likely underestimates the true population prevalence of cannabis-related harm outcomes. Third, to date, our studies have not explored variations in specific policy provisions (e.g., number of legal dispensaries, home cultivation, and consumption restrictions). Nevertheless, our studies have several strengths including the use of large, nationally and state-representative samples across multiple years and of data reported by gender, age and racial/ethnic groups.

## Conclusion

In conclusion, existing US studies have shown increases in cannabis use outcomes following the enactment of medical and recreational cannabis laws, but these increases are limited to adults. To date, no changes in the prevalence of any cannabis use or daily/near daily use have been observed among adolescents after medical or recreational legalization.^30,40,51-53^ A recent study^40^ found increases in cannabis use disorder after recreational legalization among 12 to 17-year-olds who used cannabis in the past year, from 22.8% to 27.2%; however, the authors cautioned that associations between recreational cannabis law passage and these increases could reasonably be attributed to random error or unmeasured confounding. Additional work is needed to replicate and interrogate whether this change is attributable to legalization, particularly as the prevalence of cannabis use in this age group declined from 2002-2018.^1^

Ultimately, accurate empirical evidence, rather than ideology, should guide decision-making around cannabis policies. At present, there is little evidence to suggest widespread negative effects on cannabis use outcomes following the enactment of medical or recreational cannabis laws. Epidemiologic research is ongoing on the effects of cannabis policies, as well as on variations in such policies, and longitudinal studies with longer follow-up times are sorely needed.
